# Model-Based Meta-Analysis of Relapsing Mouse Model Studies from the Critical Path to Tuberculosis Drug Regimens Initiative Database

**DOI:** 10.1128/aac.01793-21

**Published:** 2022-03-15

**Authors:** Alexander Berg, James Clary, Debra Hanna, Eric Nuermberger, Anne Lenaerts, Nicole Ammerman, Michelle Ramey, Dan Hartley, David Hermann

**Affiliations:** a Cognigen Corporation, a Division of Simulations Plus, Inc., Buffalo, New York, USA; b Bill and Melinda Gates Foundation, Seattle, Washington, USA; c Department of Medicine, Johns Hopkins University, Baltimore, Maryland, USA; d Department of Microbiology, Immunology and Pathology, Colorado State University, Fort Collins, Colorado, USA; e Erasmus MC, University Medical Center Rotterdam, Department of Medical Microbiology and Infectious Diseases, Rotterdam, the Netherlands; f Critical Path Institute, Tucson, Arizona, USA

**Keywords:** tuberculosis, relapsing mouse model, *Mycobacterium*, model-based meta-analysis, modeling and simulation

## Abstract

Tuberculosis (TB), the disease caused by Mycobacterium tuberculosis (Mtb), remains a leading infectious disease-related cause of death worldwide, necessitating the development of new and improved treatment regimens. Nonclinical evaluation of candidate drug combinations via the relapsing mouse model (RMM) is an important step in regimen development, through which candidate regimens that provide the greatest decrease in the probability of relapse following treatment in mice may be identified for further development. Although RMM studies are a critical tool to evaluate regimen efficacy, making comprehensive “apples to apples” comparisons of regimen performance in the RMM has been a challenge in large part due to the need to evaluate and adjust for variability across studies arising from differences in design and execution. To address this knowledge gap, we performed a model-based meta-analysis on data for 17 unique regimens obtained from a total of 1592 mice across 28 RMM studies. Specifically, a mixed-effects logistic regression model was developed that described the treatment duration-dependent probability of relapse for each regimen and identified relevant covariates contributing to interstudy variability. Using the model, covariate-normalized metrics of interest, namely, treatment duration required to reach 50% and 10% relapse probability, were derived and used to compare relative regimen performance. Overall, the model-based meta-analysis approach presented herein enabled cross-study comparison of efficacy in the RMM and provided a framework whereby data from emerging studies may be analyzed in the context of historical data to aid in selecting candidate drug combinations for clinical evaluation as TB drug regimens.

## INTRODUCTION

Mycobacterium tuberculosis (Mtb), the causative agent of tuberculosis (TB), infects an estimated one-quarter of the world’s population, and causes an estimated 1.4 million TB-related deaths per year, making it the leading worldwide cause of death due to a single infectious disease excluding severe acute respiratory syndrome coronavirus 2 (SARS-CoV-2) (1–3). Despite a few successes in the past decade in bringing forward new treatments for drug-resistant pulmonary TB (4–6), progress in advancing new drugs and regimens for pulmonary TB has been limited. The standard-of-care treatment regimen for drug-susceptible pulmonary TB, based on the combination of isoniazid, rifampin, pyrazinamide, and ethambutol, remains essentially unchanged for more than 30 years (7). While the regimen is efficacious (∼90% cure rates in a clinical trial setting), its complexity, long treatment duration, often poor tolerability, and requirement for high adherence to minimize treatment failure represent gaps that must be addressed through the development of novel treatment regimens. Treatment with the standard regimen is typically 6 months and may be up to 9 months in duration. The major aim of ongoing TB drug research is to develop markedly shorter treatments (e.g., less than 2 months) to improve adherence and overall cure rates.

To accelerate progress in fighting the global TB pandemic, organizations such as the Bill and Melinda Gates Foundation have worked to promote renewed interest in TB drug development through funding collaborative efforts to accelerate the development of new drug regimens. One such effort, the Critical Path to TB Drug Regimens (CPTR) Initiative, was formed to facilitate the development of new tools and methodologies for use in TB drug and regimen development (8). Since its inception, a primary focus of the CPTR Initiative has been the aggregation and standardization of clinical and nonclinical data sets to enable pooled analyses of existing data on TB drug regimens. These analyses, such as the TB-ReFLECT meta-analysis (9), provide greater insight into questions underlying the research and development of new drugs and regimens to guide the design of new studies and selection of regimens for further evaluation.

The recent uptick in investment and collaborative efforts in TB research and development has resulted in the identification of numerous drug candidates with potential for efficacy in the treatment of both drug-susceptible and drug-resistant TB. Given the multitude of potential combinations of existing and novel drugs, the prioritization of candidate regimens is now a significant challenge, especially for candidate regimens for which clinical data are not yet available for one or more regimen components. The selection of such novel regimens for further advancement into clinical studies relies heavily on the comparison of nonclinical efficacy studies (10), particularly the relative performance of regimens in achieving nonrelapsing cures. This endpoint is commonly assessed using the relapsing mouse model (RMM) (11), a murine model of TB which tests the overall curative potential of a drug combination by evaluating the proportion of mice exhibiting relapse following different treatment durations. In the RMM, relapse is defined as recurrence of Mtb growth in cultures of the lung (and sometimes spleen) tissue samples obtained postsacrifice after a posttreatment clearance period of typically three to 6 months. Although this definition of relapse implies the occurrence of no culture growth at the end of treatment, repeated tissue sampling of Mtb growth is not possible in individual mice, and operationally this is not an absolute requirement to determine the treatment duration required to prevent relapse. Comparison of regimens in the RMM is typically done by rank-ordering based on the overall proportion of relapse at various treatment durations. Depending on the effect size, regimens that exhibit lower proportions of relapse following the completion of treatment and/or similar proportions of relapse following shorter treatment durations may be considered potential improvements upon the standard of care regimens and, thus, may be considered for clinical evaluation.

Although RMM studies are a critical tool in comparing regimen performance, the model has limitations regarding study duration, design heterogeneity, and overall utility for decision-making. Designs can vary widely from lab to lab, with differences across multiple design elements such as mouse strain, bacterial strain, inoculation dose and route, recovery duration, and bacterial culture methods. These interstudy differences, not to mention regimen-specific differences in treatment duration and dose selection, contribute to the observation that the efficacy of a specific regimen in the RMM can vary widely from study to study, even within the same lab, confounding decision-making (11–13). This interstudy variability obviates the comparison of regimens across studies, which is a key limitation when attempting to prioritize regimens because logistical considerations typically limit RMM studies to the evaluation of only a limited number of regimens in a study. Moreover, the analysis of regimen performance within a given study has historically utilized conventional group-to-group statistical comparisons designed to evaluate the proportion of mice relapsing at a relatively small number of treatment durations (e.g., three to five). Lenaerts et al. (14) demonstrated that the RMM requires at least 15 animals per regimen to detect a 50% reduction in relapse probability at a given treatment duration with at least 80% power. This analytical strategy based upon a point-by-point comparison of relapsing proportions limits the utility of the model as the differences between candidate regimens are often much smaller. Further, the focus on comparative efficacy at prespecified treatment durations requires careful selection of multiple treatment durations (and larger numbers of mice) to be tested to ensure identification of regimens that achieve significant rates of cure (e.g., 10% or lower probability of relapse) with a shorter treatment duration than the current standard of care regimen.

The objective of this work, which was based on data from a total of 28 studies conducted in two separate laboratories, was to develop a suitable model-based regression approach to (i) determine the treatment duration-dependent relapse probability profiles for the included drug regimens, (ii) quantify the magnitude of interstudy variability in treatment response and assess the impact of study-level covariates on treatment response, and (iii) calculate key metrics of interest, namely, treatment duration required to reach 50% and 10% relapse probability (T_50_ and T_10_, respectively) for comparison of relative regimen performance in the RMM when adjusted for study-level covariates. By applying a model-based analysis, it was expected that interstudy differences could be accounted for and unbiased estimates of informative parameters, such as T_10_, could be reported to support decision-making. Importantly, the model-based approach was also expected to yield uncertainty in parameters, such as T_10_, which will be a function of the amount of and consistency in available data, further contributing to informed decision-making.

## RESULTS

### Exploratory data analysis.

Data from 1310 mice were included in the first stage of the analysis of 25 studies, with data from three more studies contributing an additional 282 mice added in the second stage, for a total of 1592 mice across 28 studies. Summary statistics across all studies in the analysis are shown in Table 1, with a further summary of data for each regimen provided in Table 2.

**TABLE 1:** Summary statistics for study-level data

Variables	Statistics
Total no. of mice	Median: 45 mice Range: 13–165 mice
Total no. of regimens	Median: 1.5 regimens Range: 1–6 regimens
Treatment duration	Median: 4 mo Range: 2–9 mo
Mice per time point per regimen	Median: 15 mice Range: 8–41 mice
The lower limit of detection on solid media	Median: 0.3 log_10_ CFU Range: 0.3–1.18 log_10_ CFU
Plated lung portion	Median: 1.0 Range: 0.07–1.00
Infection duration before treatment	Median: 19 days Range: 13–56 days
Culture incubation duration	Median: 37 days Range: 20–62 days Missing: 5.6%
Inoculum size	Median: 3.29 log_10_ CFU Range: 1.27–4.66 log_10_ CFU Missing: 6.4%
Baseline bacterial burden	Median: 6.79 log_10_ CFU Range: 5.50–9.02 log_10_ CFU
Recovery duration posttreatment	12 wks (92%) 24 wks (8%)
Media type	7H10 (9.5%) 7H11 (26%) 7H11 selective (55.9%) 7H11 with AmphoB or cyclohexadiene (0.9%) 7H11 selective with charcoal (7.7%)
Fixed dosing (FIX)	Time-variable mg/kg dose (13.4%) Fixed mg/kg dose (86.6%)
Inoculation groups	1 group (5.6%) 2 groups (52.7%) 3 groups (18.3%) 4 groups (23.4%)
Contributing laboratory	CSU^*a*^ (26%) JHU^*a*^ (74%)

a CSU, Colorado State University; JHU, Johns Hopkins University.

**TABLE 2:** Summary of available data by regimen

Regimen^*a*^	No. of studies	No. of mice	Assessed treatment durations (months)
BPa	3	79	2, 3, 4
BPaL	4	139	2, 3, 4
HE	1	30	6, 9
HRE	1	31	3, 6
HRE/HR	1	45	3, 4.5, 6
HRZ/HR	15	531	2, 2.5, 3, 4, 5, 6
HRZE	5	105	2, 3, 4
HRZE/HR	9	199	3, 4, 4.5, 5, 6
HRZE/HRZ	1	15	3
HRZM	2	40	2, 3
HRZM/HRM	2	23	3, 6
HZE	1	29	3, 6
RMZ	1	36	3, 4, 5
RMZ/RM	5	215	3, 4, 5, 6
RMZ/RM^*b*^	1	15	2.5
RMZE	2	45	2, 3
RMZE/RM	1	15	3

a B, bedaquiline; E, ethambutol; H, isoniazid; L, linezolid; M, moxifloxacin; Pa, pretomanid; R, rifampin; Z, pyrazinamide.

b Moxifloxacin 100 mg/kg dosed twice daily for a total daily dose of 200 mg/kg.

**FIG 1:**
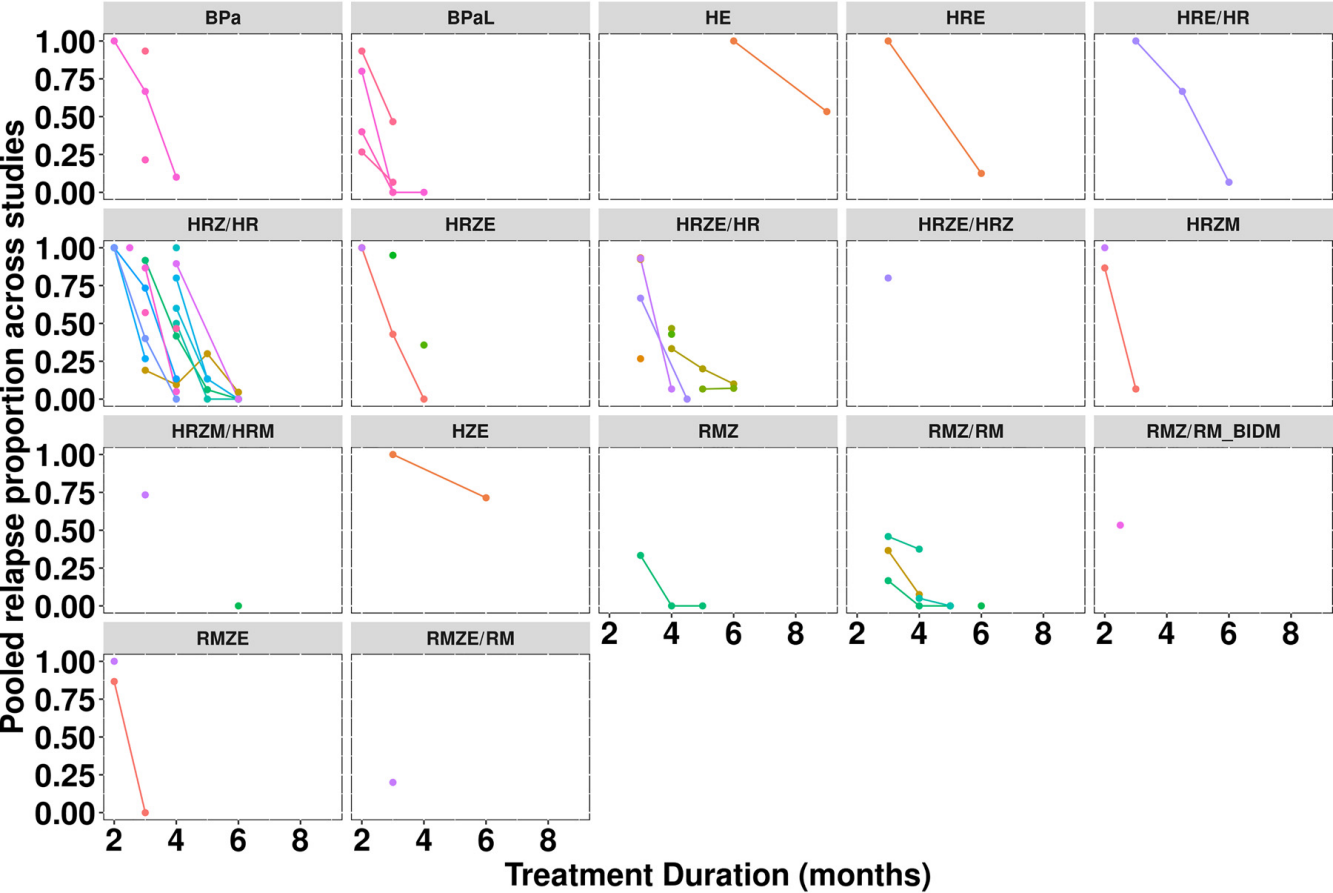
Relapse proportion by treatment duration across stratified by regimen and study. Each study is grouped with lines representing the relapse-time course in a particular study. Points represent individual time points in a particular study. “RMZ/RM_BIDM” denotes a version of the RMZ/RM regimen where moxifloxacin was administered at 100 mg/kg twice daily for a total daily dose of 200 mg/kg. M, moxifloxacin; R, rifampin; Z, pyrazinamide.

Observed relapse proportions for each regimen by treatment duration are shown in Fig. 1, where results are grouped by study and stratified by contributing laboratory. Significant variability in response was observed across studies, which was visible during exploratory analysis for the most utilized control regimen, HRZ/HR . As judged by the graphic depiction of raw data, the HRZ/HR treatment duration to reach 50% relapse probability (i.e., 50% proportion of mice exhibiting relapse) ranged from less than 2.5 months to greater than 4.5 months. This observation is consistent with previous observations of variable treatment response for regimens across studies by using different mouse models (12) and is expected given the differences in study design and covariates. To quantify the magnitude of the interstudy variability and investigate which covariates may be potential sources of variability, a mixed-effects logistic regression modeling approach was applied under the assumption of logit-linearity (confirmed before analysis; Fig. S1).

### Model development and evaluation.

To improve the overall utility of RMM study data and interpretation for regimen prioritization, the CPTR Initiative applied modeling and simulation-based techniques to the analysis of RMM study data across multiple pooled, historical data sets. Key steps in model development are summarized in Table 3, which lists the pivotal model runs from the primary analysis stage. As expected, the inclusion of treatment-specific intercept (INT) and slope (SLP) terms greatly improved the model fit compared to a “naïve” model, including shared INT and SLP terms across all treatments and study as a random effect on both INT and SLP. Subsequent model simplification to group similar treatments to improve model stability before covariate analysis further reduced the Akaike’s information criterion (AIC) value of the model. Stepwise covariate analysis resulted in the identification of inoculum amount (INOC) and average baseline lung bacterial burden (BASE) as significant covariates on INT and SLP, respectively. This further reduced both the objective function value (OFV) and the AIC values and eliminated trends in goodness-of-fit plots of random effects (η_j_ values) versus covariates (not shown). Further, the inclusion of these covariates accounted for a significant amount of interstudy variability, decreasing the interstudy standard deviation estimates for INT (ω_INT_) and SLP (ω_SLP_) by 37% and 23%, respectively. Subsequent model refinements during the primary analysis phase focused on separating INT and/or SLP terms for treatment regimens that were grouped before covariate analysis while maintaining model stability. This was done to support our primary objective of extracting treatment duration-dependent relapse probability profiles from the model to assess differences in response between regimens and resulted in further model improvements. In the second stage of the analysis, the model was updated to support the inclusion of additional data sets that became available during the project. This update included the addition of treatment-specific INT and SLP terms for the BPa and BPaL regimens not included in the original data set but did not result in any further modification of the model structure. Goodness-of-fit plots for the final model are presented in Fig. S2. The final parameter-covariate relationships are shown below:

(1) $$\mathrm{IN}T_{i,j}=\mathrm{IN}T_{\mathrm{TRT}}+1.4\times (\mathrm{INO}C_{j}-3.29)+\eta_{\mathrm{INT},j}$$

(2) $$\mathrm{SL}P_{i,j}=\mathrm{SL}P_{\mathrm{TRT}}+0.497\times (\mathrm{BAS}E_{j}-6.79)+\eta_{\mathrm{SLP},j}$$

**TABLE 3:** Listing of pivotal models during the primary analysis phase

Model no.	Description	OFV^*a*^	AIC^*b*^
3	Naïve model	1131.2	1141.2
25	“Full” treatment effect, no covariates	987.1	1033.1
35	“Reduced” treatment effects, no covariates	990.1	1018.1
37	Reduced treatment effects plus covariates	899.2	931.2
44	Final model	882.4	920.4

a OFV, objective function value.

b AIC, Akaike’s information criterion.

Estimates for the treatment-specific fixed-effects parameters are presented in Table 4, whereas random-effects estimates from the final model were 1.209 and 0.636 for ω_INT_ and ω_SLP_, respectively, with an estimated correlation of −0.75.

**TABLE 4:** Treatment-specific fixed-effects parameter estimates

Regimen^*a*^	INT_TRT_		SLP_TRT_	
	Estimate	SE	Estimate	SE
BPa	2.270	1.110	−1.96	0.892
BPaL	0.499	0.780	−3.77	0.782
HE	21.50	2.420	−3.00	0.270
HRE or HRE/HR	9.580	0.998	−3.00	0.270
HRZ/HR	4.860	0.481	−3.00	0.270
HRZE or HRZE/HRZ	4.700	0.438	−3.23	0.317
HRZE/HR	4.700	0.438	−3.00	0.270
HRZM	3.610	0.759	−4.79	1.260
HRZM/HRM	3.610	0.759	−3.00	0.270
HZE	12.70	1.330	−3.00	0.270
RMZ/RM_BIDM^*b*^	0.654	1.160	−3.11	0.492
RMZ	2.220	0.885	−3.54	0.787
RMZ/RM	2.220	0.885	−3.11	0.492
RMZE	2.110	0.771	−3.54	0.787
RMZE/RM	2.110	0.771	−3.11	0.492

a B, bedaquiline; E, ethambutol; H, isoniazid; L, linezolid; M, moxifloxacin; Pa, pretomanid; R, rifampin; Z, pyrazinamide.

b Moxifloxacin 100 mg/kg dosed twice daily for a total daily dose of 200 mg/kg.

**FIG 2:**
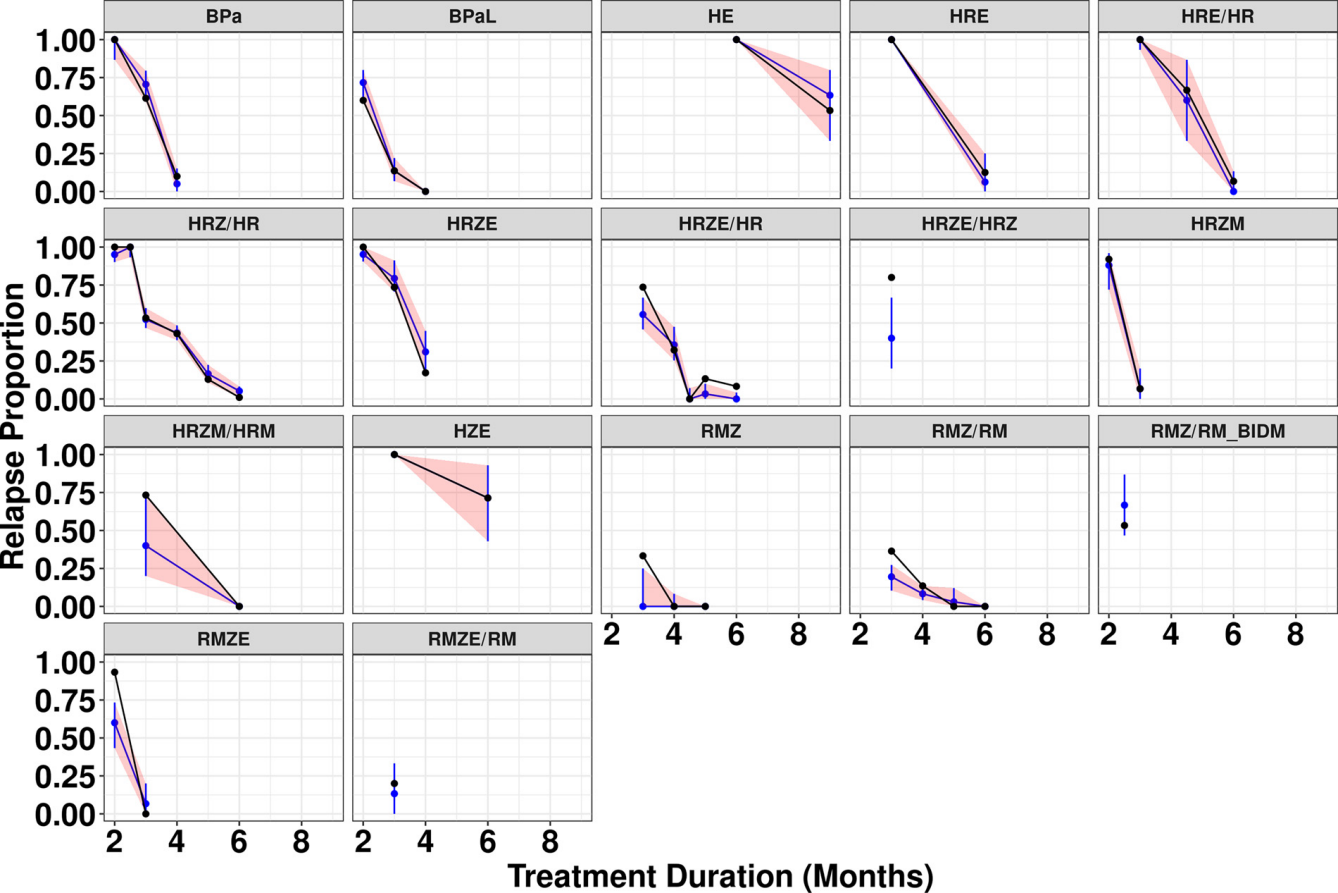
Visual predictive check for the final model stratified by regimen. Black dots and solid lines represent the observed relapse proportion. Blue lines represent the median prediction from the final model. The red shaded area represents the 90% prediction interval. “RMZ/RM_BIDM” denotes a version of the RMZ/RM regimen where moxifloxacin was administered at 100 mg/kg twice daily for a total daily dose of 200 mg/kg. M, moxifloxacin; R, rifampin; Z, pyrazinamide.

To assess the predictive capability of the final model, a visual predictive check (VPC) was performed with stratification by treatment (Fig. 2). Observed relapse proportions were generally within the 95% prediction interval for the various regimens, although underprediction was observed at a single treatment duration for a few regimens. Additional VPCs with stratification by study-specific covariate values for INOC and BASE as well as by study (Fig. S3 to S5) show a similar pattern of agreement between observed and predicted values. Taken together, the VPCs indicate that the model exhibits acceptable predictive performance.

### Comparison of regimen efficacy.

**FIG 3:**
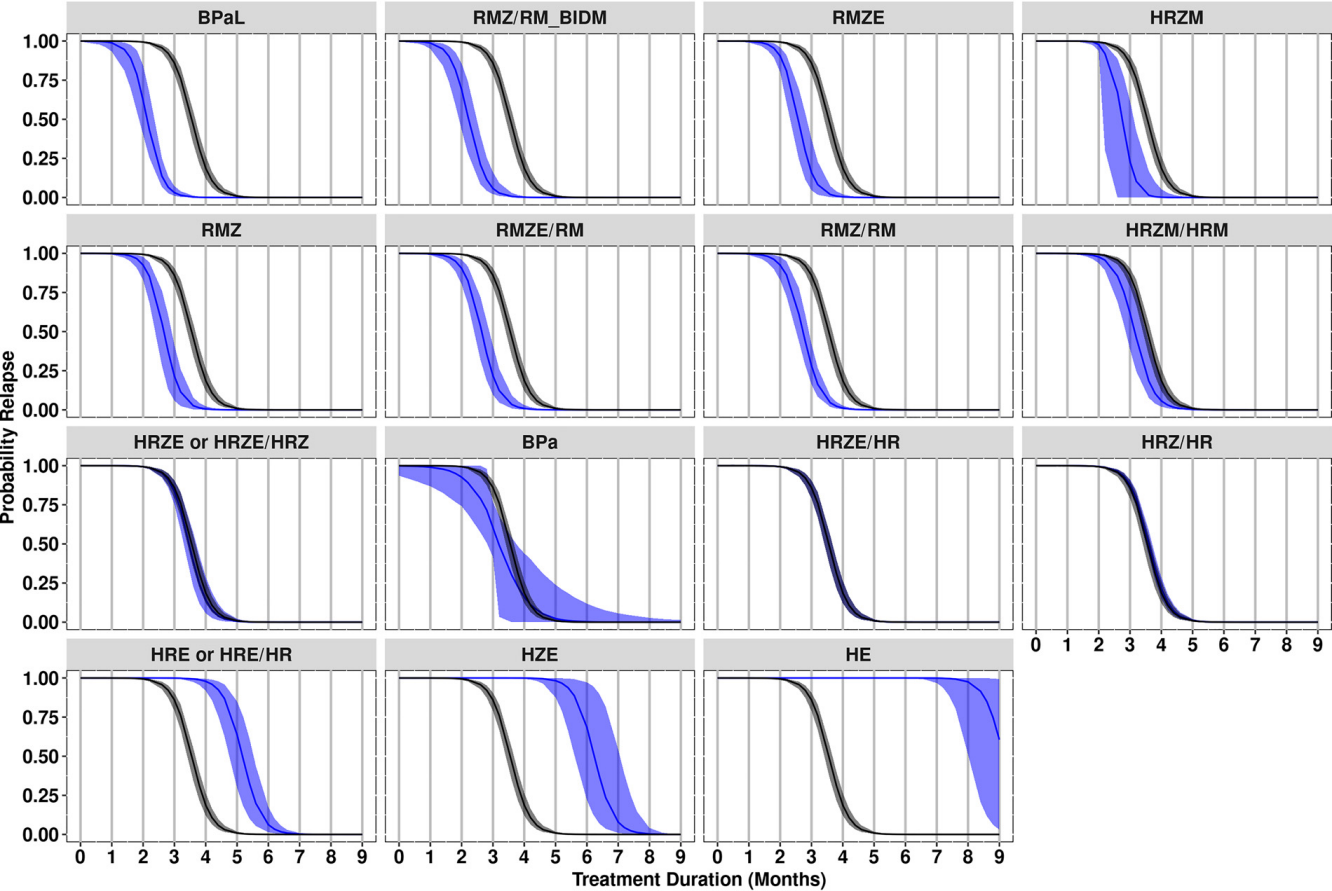
Relapse probability versus treatment duration by regimen. Blue lines and areas represent the median and 95% confidence interval, respectively, of relapse probability versus treatment duration profiles for each regimen as derived by bootstrap (N = 500 runs). Black lines and areas represent HRZE/HR as the clinical standard of care regimen. For comparative purposes, all regimens are presented as normalized to the median covariate values. “RMZ/RM_BIDM” denotes a version of the RMZ/RM regimen where moxifloxacin was administered at 100 mg/kg twice daily for a total daily dose of 200 mg/kg. E, ethambutol; H, isoniazid; M, moxifloxacin; R, rifampin; Z, pyrazinamide.

**FIG 4:**
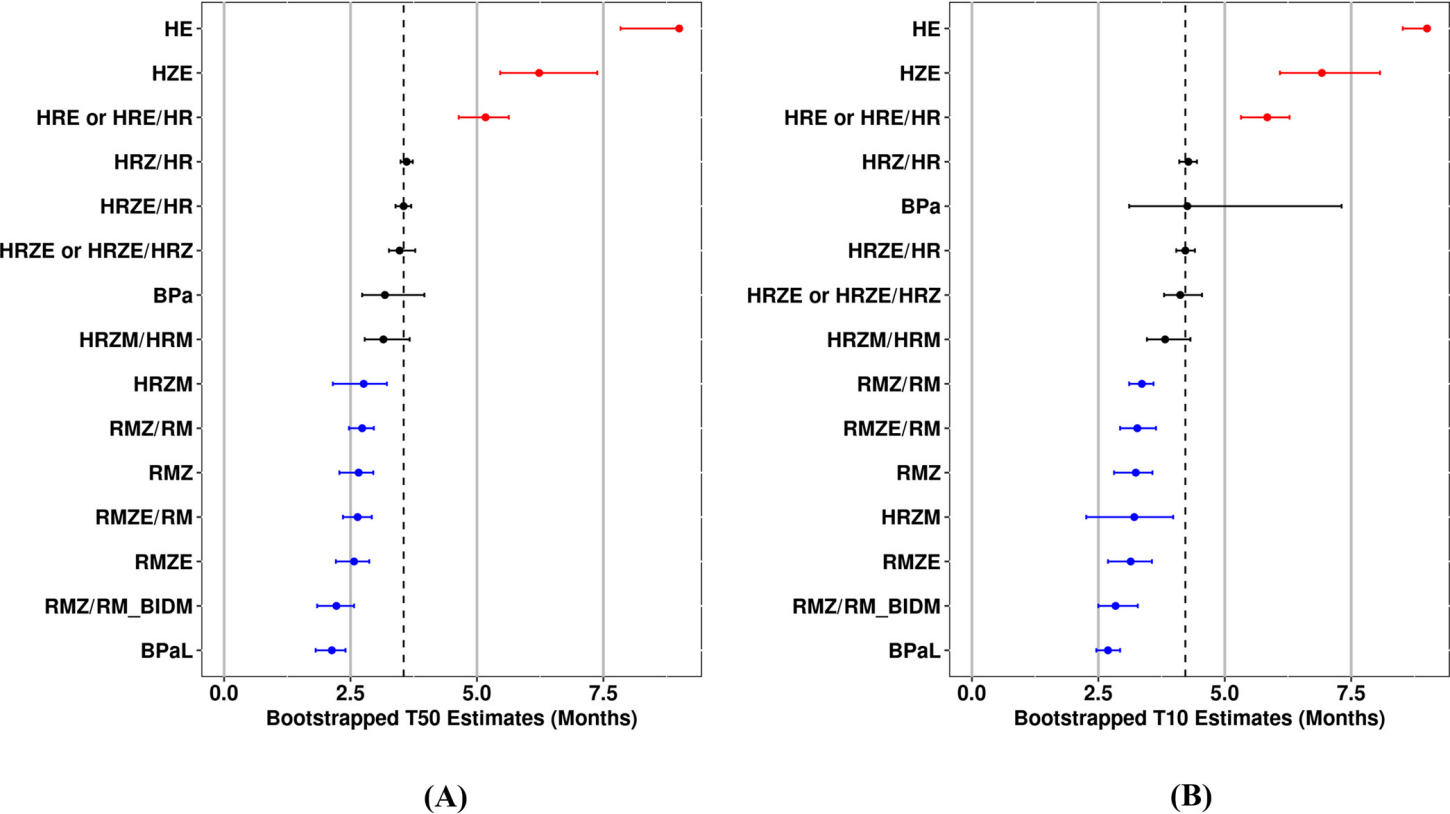
Forest plots of T_50_ and T_10_ relapse probability by regimen. Median estimates and 95% confidence intervals from bootstrapped data sets (*n* = 500 runs) for (A) T_50_ and (B) T_10_ metrics. Regimens are in descending rank orders for metrics based on median value, with red and blue coloring indicating regimens for which the confidence intervals are completely above or below the median value for HRZE/HR (included as the clinical standard of care regimen). For comparative purposes, all regimens are presented as normalized to the median covariate values. “RMZ/RM_BIDM” denotes a version of the RMZ/RM regimen where moxifloxacin was administered at 100 mg/kg twice daily for a total daily dose of 200 mg/kg. E, ethambutol; H, isoniazid; M, moxifloxacin; R, rifampin; Z, pyrazinamide.

A bootstrap analysis was performed to compare the efficacy of the various treatment regimens and obtain distribution-independent precision estimates. Relapse probability versus treatment duration profiles derived at the covariate median values using estimates obtained from each bootstrap replicate are shown in Fig. 3 along with HRZE/HR as the clinical standard of care regimen. Covariate-normalized T_50_ and T_10_ values were also obtained from each bootstrap replicate and are depicted in Fig. 4A and B, respectively, with median and 95% confidence intervals (CIs) and regimen rank order based on the median values provided in Table 5. As observed in Fig. 3, efficacy profiles are generally well estimated for most treatment regimens, although BPa exhibited a relatively large confidence interval attributed to poorer precision in the BPa-specific SLP parameter secondary to the relatively small number of treatment durations and mice available for this regimen. This is consistent with the forest plots, which show that the T_10_ confidence interval for BPa is much wider for this regimen compared to the other regimens. Although T_10_ generally showed a slightly broader confidence interval for all regimens compared to T_50_, both parameters showed the same three groupings based upon whether their confidence interval overlapped the median estimate for HRZE/HR: (i) regimens with better performance (HRZM, RMZ/RM, RMZ, RMZE/RM, RMZE, and BPaL), (ii) regimens with similar performance (HRZ/HR, HRZE, HRZE/HRZ, BPa, and HRZM/HRM), and (iii) regimens with poorer performance (HE, HRE, HRE/HR, and HZE). Similarly, rank-ordering demonstrated that BPaL was the best performing regimen for both T_10_ and T_50_ values (median values of 2.13 months and 2.69 months, respectively), with ranks for other regimens also consistent except for HRZM and BPa (ranked 4th and 11th, respectively, based on T_10,_ and 7th and 9th, respectively, based on T_50_).

**TABLE 5:** Regimen-specific T_50_ and T_10_ estimates in months and corresponding rank-order

Regimen^*a*^	Model estimate	Bootstrap median	Bootstrap 95% confidence interval	Rank order per bootstrap median
Time to 50% relapse				
BPaL	2.13	2.13	1.81 – 2.40	1
RMZ/RM_BIDM^*b*^	2.21	2.22	1.84 – 2.57	2
RMZE	2.60	2.57	2.21 – 2.87	3
RMZE/RM	2.67	2.64	2.35 – 2.92	4
RMZ	2.63	2.66	2.28 – 2.95	5
RMZ/RM	2.71	2.73	2.47 – 2.96	6
HRZM	2.75	2.76	2.15 – 3.22	7
HRZM/HRM	3.20	3.15	2.78 – 3.67	8
BPa	3.16	3.18	2.73 – 3.96	9
HRZE or HRZE/HRZ	3.46	3.47	3.26 – 3.78	10
HRZE/HR	3.57	3.55	3.39 – 3.7	11
HRZ/HR	3.62	3.61	3.49 – 3.73	12
HRE or HRE/HR	5.19	5.17	4.64 – 5.63	13
HZE	6.23	6.23	5.46 – 7.38	14
HE	≥9.00	≥9.00	7.84 – ≥9.00	15
Time to 10% relapse				
BPaL	2.72	2.69	2.46 – 2.93	1
RMZ/RM_BIDM^*b*^	2.92	2.84	2.50 – 3.28	2
RMZE	3.22	3.14	2.69 – 3.56	3
HRZM	3.21	3.21	2.26 – 3.98	4
RMZ	3.24	3.24	2.81 – 3.57	5
RMZE/RM	3.38	3.27	2.93 – 3.64	6
RMZ/RM	3.42	3.36	3.11 – 3.59	7
HRZM/HRM	3.94	3.82	3.46 – 4.32	8
HRZE or HRZE/HRZ	4.14	4.12	3.80 – 4.55	9
HRZE/HR	4.30	4.22	4.04 – 4.41	10
BPa	4.28	4.26	3.11 – 7.31	11
HRZ/HR	4.35	4.28	4.10 – 4.45	12
HRE or HRE/HR	5.93	5.84	5.32 – 6.28	13
HZE	6.97	6.92	6.09 – 8.07	14
HE	≥9.00	≥9.00	8.52 – ≥9.00	15

a B, bedaquiline; E, ethambutol; H, isoniazid; L, linezolid; M, moxifloxacin; Pa, pretomanid; R, rifampin; Z, pyrazinamide.

b Moxifloxacin 100 mg/kg dosed twice daily for a total daily dose of 200 mg/kg.

## DISCUSSION

Historically, results from RMM studies have been reported in raw data tables with limited supporting statistical analyses limiting comparisons, interpretation, and decision-making across RMM studies and regimens. Through the application of model-based meta-analysis approaches to a large data set of 28 studies, we have been able to improve the understanding and interpretability of regimen performance in the relapsing mouse model of TB infection. Model-based analyses have a distinct advantage over standard statistical methods used to analyze RMM studies in that the underlying mathematical model allows researchers to compare regimens not solely based on the proportions of mice in a treatment group relapsing at prespecified treatment durations, but rather to obtain the relapse probability at any treatment duration (within the approximate range of durations experimentally explored). We accomplished this in the present study through a relatively simple logistic regression approach, which utilized observed binary relapse data for individual mice to estimate the INT and SLP parameters that describe the logit-linear relationship between relapse probability and treatment duration for each regimen. It is noted that this is analogous to that applied previously by Mourik et al. (15) and, more recently, by Mudde et al. (16), which utilized a sigmoid maximum effect (E_max_) model. Although based on slightly different assumptions, both mathematical models enabled the calculation of model-based parameters of interest and the derivation of continuous relapse probability versus treatment duration profiles, as seen in Fig. 3. Such model-based outputs are highly informative when interpreting regimen performance in RMM studies and enable quantitative comparisons to control regimens based on metrics, such as the T_10_ that was analogous to clinically relevant endpoints of interest (i.e., treatment duration required to achieve an acceptably low cure rate).

**FIG 5:**
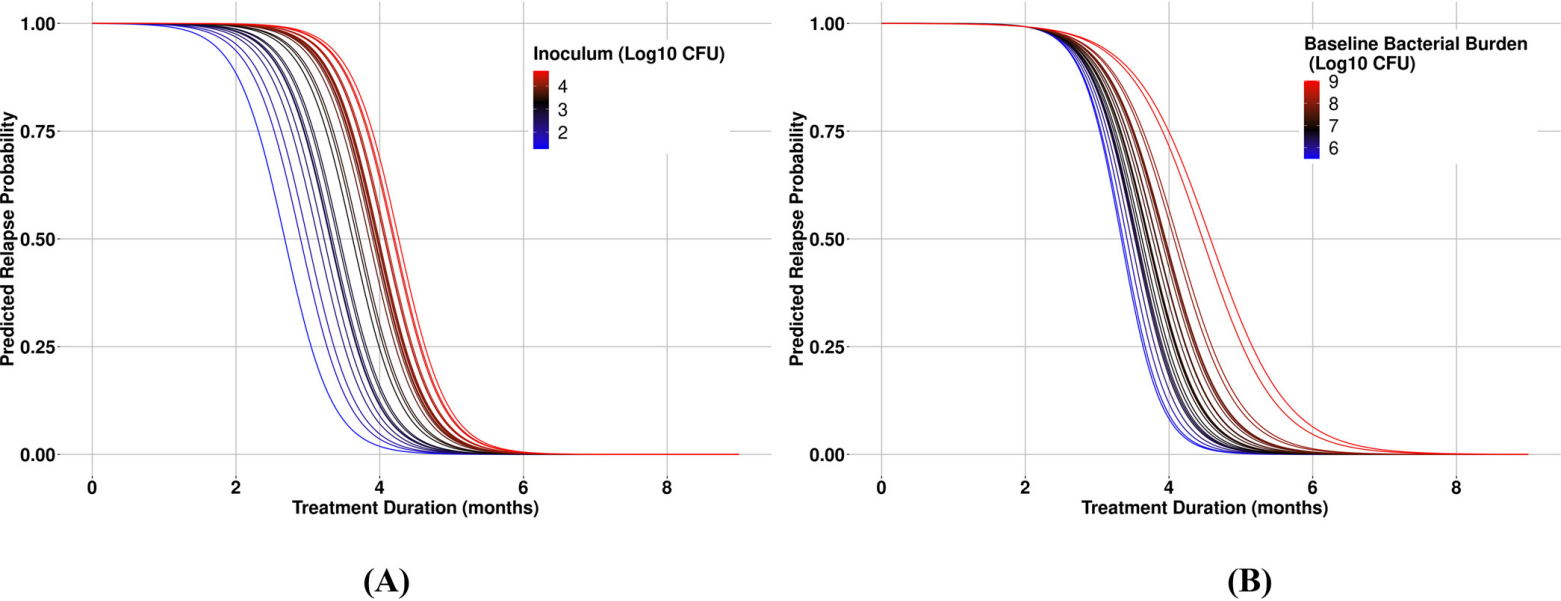
Simulations of the model with a range of covariate effects. Simulations of covariate effects from range of data for (A) inoculum (log_10_ CFU/mL) and (B) baseline bacterial burden (log_10_ CFU/mL).

Aside from the similarity in the underlying mathematical models, the present analysis extends beyond that of models based on single relapse studies (15, 16). Given the significantly larger data set of more than 1500 mice from 28 studies, the present model-based meta-analysis methodology was able to account for interstudy covariate and random effects on the INT and SLP parameters. An advantage of this mixed-effects modeling approach was that it partitioned the observed variability in the data into interstudy variability and residual variability, which enabled quantification of interstudy standard deviations for INT and SLP as well as exploration of study-level variables as possible sources of the observed interstudy variability. The two significant covariates identified during model development as being significant contributors to interstudy variability, inoculum amount (INOC) and baseline lung bacterial burden (BASE), accounted for more than 37% and 23% of the observed interstudy variability in INT and SLP, respectively. The effect of each covariate on the overall relapse probability versus treatment duration profile is illustrated in Fig. 5 for HRZE/HR under the assumption of no interaction between covariate effects and treatment. In both cases, as the covariate value increases, the curve shifts to the right, thereby increasing the probability of relapse at a given treatment duration. This is intuitive, as it has been demonstrated that administration of a higher inoculum of Mtb to BALB/c mice results in a greater bacterial burden in the lung, a more severe infection, and a longer treatment duration required to prevent relapse (17). Given the correlation between inoculum size and bacterial burden at treatment start (Pearson correlation = 0.46), the joint effect estimated for HRZE/HR across the observed combinations of these variables ranges from 2.68 to 4.58 months for T_50_ and 3.41 to 5.74 months for T_10_. Hence, the potential influence on study results is significant and highlights the importance of accounting for these interstudy sources of variability when considering both the design (i.e., controlling the inoculum size) as well as in the analysis and interpretation of the RMM study data.

The ability to account for study-level covariates and quantify interstudy variability highlights a further advantage to the model-based meta-analysis methodology applied herein, in that unbiased regimen-specific parameters were estimated simultaneously across data from all studies. Specifically, the treatment regimen fixed-effects parameters correspond to the covariate-normalized relapse probability versus treatment duration profiles after adjustment for residual interstudy variability. This is significant in that it allows for robust “apples-to-apples” comparisons of all regimens in the data set, despite many not being evaluated together in the same experiment. That is important considering the T_50_ estimates presented elsewhere for certain regimens (15, 16), as the impact of the significant covariates identified herein as well as other influential covariates that may not be known at this time, must be considered for cross-study comparisons. In this regard, it is noted that the ability to make cross-study comparisons within this meta-analysis were bolstered by the presence of reference regimens (e.g., HRZ/HR), which helped to partition interstudy variability from treatment effects, especially for those regimens for which data were available from only a single study or where the number of treatment durations was limited. In the latter case, even with a reference regimen “anchor,” model stability often necessitated grouping similar regimens together to share an identical fixed-effect term for either INT or SLP. Groupings are indicated in Table 4, although it is noteworthy that multiple regimens did not share the same fixed effects for both parameters except for HRE and HRE/HR as well as HRZE and HRZE/HRZ. Hence, aside from these regimens which are treated as identical (which is reasonable based upon their similarity, being differentiated only on the presence and absence of ethambutol beyond 2 months treatment duration), the structure of the final model enabled the calculation of regimen-specific relapse probability versus treatment duration profiles.

Regarding relative regimen efficacy, the results in Fig. 3 to 4 and Table 5 illustrated that, after adjusting for covariate values and interstudy variability, trends in regimen efficacy follow expected patterns. For example, the established significance of rifampin and/or pyrazinamide in shortening the treatment duration required to prevent relapse with the standard of care HRZE regimen is observed by the stepwise decreases in the median T_10_ value from HE > HZE > HRE > HRZE. Similarly, the lack of differentiation between HRZ/HR and HRZE/HR supports the generally accepted premise that ethambutol adds little to the efficacy of the HRZ-based regimen in the BALB/c RMM. The treatment shortening effect of fluoroquinolone-based regimens is also clearly seen, with median T_10_ values between 0.40 months (HRZM/HRM) and 1.38 months (RMZ/RM [twice-daily 100 mg/kg moxifloxacin]) shorter than HRZE/HR. This is notable in that the treatment-shortening effect seen in initial/early mouse studies was a consideration in advancing fluoroquinolone-containing RZ-based regimens into clinical trials with a 4-month treatment duration compared to the 6-month treatment duration for the standard of care HRZE/HR regimen (18, 19). However, even under the optimistic assumption of a direct one-to-one translation of relative regimen performance from mice to humans, the efficacy estimates obtained from our model suggest that fluoroquinolone-containing RZ-based regimens may only be capable of shortening treatment duration by approximately 1 month, consistent with the conclusions reached by Li et al. and Wallis et al. (20, 21). These findings are also in line with the position outlined by Lanoix et al., that the failures seen in phase 3 trials of 4-month fluoroquinolone-containing RZ-based regimens do not reflect the poor predictive performance of the RMM but rather an overly optimistic translation of RMM findings for these regimens to the clinic (22). It should be noted, however, that the treatment-shortening effect of fluoroquinolones is dependent upon the overall drug combination administered because the combination of moxifloxacin, rifapentine, isoniazid, and pyrazinamide has recently been reported as an effective 4-month regimen (23). Overall, our findings demonstrate that model-based analysis of RMM data provides results that are not only consistent with previous studies but also build on the understanding and interpretability of RMM studies by providing robust, quantitative, and meaningful measures of relative regimen efficacy.

Although the model developed in the current study is limited to only those regimens included in the historical CPTR data set, the model itself is not “static” and allows for continuous updating to include data from new studies and regimens and may be applied to inform the design of future RMM studies (through *in silico* trial simulation). In the latter, further iterations of the model are being developed using emerging data on new regimens to assess the contribution of various regimen components and help select promising regimens for future study. An example of this approach is described in this report where the model was updated through the incorporation of additional data for the novel BPa and BPaL regimens. The model-based estimates show that the novel two-drug BPa backbone is as efficacious in the RMM as the standard of care regimen and that the addition of linezolid as a third component provides significant improvement in regimen efficacy (Table 5), with the resulting BPaL regimen being superior to HRZE/HR. Of importance, though, the model-based estimates indicate that BPaL may be no better than fluoroquinolone-based regimens at shortening overall treatment duration. Consequently, while BPaL is a highly efficacious regimen, it may not be capable of meeting the goal of treatment shortening to less than 2 months. Prioritization of new regimens should be informed using the present model-based approach through the accumulation of new RMM data on new candidate regimens pooled with the existing historic data set, and subsequent model reestimation. Multiple RMM studies are currently planned or ongoing that will generate additional data, with the design of several studies directly informed using the current model. Specifically, the mixed-effects logistic regression model has been applied to undertake RMM trial simulations to evaluate attributes, such as number of mice per arm, number of treatment durations, inoculum size, different hypothetical treatment regimens, and inclusion of control arms. The relapse outcomes of simulated “virtual” mice are then analyzed using the same model-based approach to assess the performance of the various designs in terms of overall bias and precision on T_50_ and T_10_. This approach, which has been highly instrumental in selecting and refining the design of RMM studies to increase precision while minimizing the number of mice required for each study, will be detailed in a subsequent report.

Although limitations of the analyses have been identified throughout this report, a few key limitations are worthy of further acknowledgment. The data used in these analyses were obtained from only two laboratories, which likely impacted the estimated interstudy variability estimate and limited the ability to evaluate certain covariates which were cofounded by the lab covariate (for example, Mtb strain). The inclusion of more data from additional laboratories will help to refine this estimate and may result in the identification of inter-laboratory differences. We also note the relatively small amount of data available for some regimens, which may have been evaluated in a single study, at limited time points, or in small numbers of mice (as low as 15 animals total). While the impact of regimen representation in a small number of studies is compensated by the estimation of covariate effects and the inclusion of a study-level random effect, regimens with low numbers of relapse assessments and/or mice are not as precisely estimated. The collection of additional data will help to improve the precision of the estimates, which may readily be incorporated as data become available through iterative model updates. Lastly, we emphasize that any inferences regarding the clinical use of these regimens, based on the results presented herein, should be made cautiously because the translation of findings from the RMM to predict clinical outcomes requires consideration of multiple factors that are not addressed by these analyses.

Taken together, the model-based meta-analysis presented herein represents an improvement in the analysis, understanding, and interpretability of data from relapsing mouse model studies. By adjusting for key study-level differences and accounting for interstudy variability, this approach generates robust, quantitative, and relevant metrics of interest, such as T_50_ and T_10_, respectively, that enhance the understanding and interpretation of RMM study data and ultimately support decision making about regimen selection and prioritization.

## MATERIALS AND METHODS

### Data.

The analysis was conducted based on data from studies conducted by the laboratories of Anne Lenaerts (Colorado State University, CSU), Jacques Grosset, and Eric Nuermberger (Johns Hopkins University, JHU). The experimental details of the contributed data sets have been described elsewhere (13, 20, 24–31). Data sets corresponding to the 25 studies were standardized to a data template developed by the authors as part of the CPTR Initiative before aggregation into the pooled analysis data set for initial model development. Data from three additional JHU studies were subsequently added to the pooled data set, resulting in a total of 28 studies in the final data set (Table S1). All studies were performed in BALB/c mice inoculated via aerosol inhalation with either Mtb Erdman TMC 107 (ATCC 35801) (CSU) or mouse passaged H37Rv (ATCC 27294) (JHU) Mtb strains. In all studies, the primary endpoint for each mouse was relapse as a binary (0/1) variable, determined based on whether Mtb growth was present or absent upon culturing a homogenate of lung tissue on solid media. Data from untreated control animals were excluded from the analysis.

A total of 17 unique regimens were available for analysis, including the following drugs and doses: bedaquiline (BDQ, B) 25 mg/kg, ethambutol (EMB, E) 100 mg/kg, isoniazid (INH, H) 10 mg/kg or 25 mg/kg, linezolid (LZD, L) 100 mg/kg, moxifloxacin (MXF, M) 100 mg/kg, rifampin (RIF, R) 10 mg/kg, pretomanid (Pa) 100 mg/kg, and pyrazinamide (PZA, Z) 150 mg/kg. Treatments were administered orally once per day on a 5 out of 7 days per week dosing schedule in all studies, except for one study that dosed moxifloxacin 100 mg/kg twice daily for a total daily dose of 200 mg/kg. Regimen designations were assigned based on standard abbreviations for regimen components with regimens containing an initial 2-month (8-week) intensive phase followed by a separate continuation phase delineated by a “/” according to the convention (i.e., HRZE/HR). Treatment duration in months was included as the independent, continuous variable for the analysis.

Study-level covariates included in the analysis data set included categorical variables (i.e., 12-week versus 24-week recovery duration posttreatment, type of solid culture media used to assess Mtb growth, fixed versus time-variable dosing, number of inoculation groups) and continuous variables (i.e., culture incubation period [days], inoculum size for aerosol [log_10_ colony forming unit (CFU)], infection duration before treatment [days], lower limit of detection for bacterial growth [log_10_ CFU], and average baseline lung bacterial burden as determined in control animals at start of treatment [log_10_ CFU]). An additional categorical “Lab” covariate designating the contributing laboratory was included due to the high degree of within-lab correlation across studies of the following variables, which were excluded from analysis: average mouse age, Mtb strain, and Mtb cultivation method for inoculum preparation. Data imputation was not performed except for one study missing the inoculum size which was therefore imputed as the median value from the data set.

### Model development.

The model presented herein was developed in two stages, with the first stage representing primary model development based on the initial data set of 25 studies, which was subsequently followed by a second stage whereby the model was updated based on data from three additional studies.

During each stage, exploratory data analysis was performed to evaluate the informational content of the data set and assess consistency across studies. Independent, dependent, and treatment variables were explored as well as relevant covariate information. Graphical outputs were used to help determine significant trends in covariates to aid in the model-building process.

Model-based analysis was performed using a generalized linear mixed-effects modeling approach. Specifically, a logistic regression model was developed with relapse treated as a binary 0 or 1 endpoint corresponding to absence or presence of relapse, respectively, treatment duration as an independent variable, and study as a random effect. Determination of model performance during development and refinement was based on standard goodness-of-fit plots and summary statistics, as well as evaluation of log likelihood-based metrics (i.e., OFV and AIC) (32). The general model structure is described by Equations 3 to 5:

(3) $$\mathrm{logit}\left(p_{i,j,\;k}\right)=\;\mathrm{IN}T_{i,j}+\mathrm{SL}P_{i,j}*(\mathrm{TIM}E_{k}-2)$$

(4) $$\mathrm{IN}T_{i,j}=\mathrm{IN}T_{\mathrm{TRT}}+\mathrm{IN}T_{\mathrm{CAT}}+\mathrm{IN}T_{\mathrm{CONT}}+\eta_{\mathrm{INT},j}$$

(5) $$\mathrm{SL}P_{i,j}=\mathrm{SL}P_{\mathrm{TRT}}+\mathrm{SL}P_{\mathrm{CAT}}+\mathrm{SL}P_{\mathrm{CONT}}+\eta_{\mathrm{SLP},j}$$

Where *p_i,j,k_* is the probability of relapse for a given treatment/covariate combination *i* in study *j* at treatment duration *k; INT_i,j_* is the intercept of the logit relapse probability after two months of treatment for the *i*th regimen in the *j*th study with a study-level covariate effect; *SLP_i,j_* is the slope of the logit relapse probability versus treatment duration for the *i*th regimen in the *j*th study with a study-level covariate effect; *TIME* is the duration of treatment for the *i*th regimen; TRT is a categorical treatment indicator (0 or 1 for the absence or presence of the regimen, respectively); CAT denotes a categorical covariate effect; CONT denotes a continuous covariate effect; η is the random effect of the *j*th study for intercept and slope, assumed to be N(0,ω^2^).

Metrics of interest, namely, T_10_ and T_50_, were calculated as follows from the model estimates according to Equations 6 and 7:

(6) $$T_{10}=\;2\;\mathrm{months}+\frac{\log\frac{0.1}{\left(1-0.1\right)}-\mathrm{IN}T_{i,j}}{\mathrm{SL}P_{i,j}}$$

(7) $$T_{50}=\;2\;\mathrm{months}-\frac{\mathrm{IN}T_{i,j}}{\mathrm{SL}P_{i,j}}$$

Treatment-specific intercept (INT) and slope (SLP) values (*viz.*, *INT_i_* and *SLP_i_*) were estimated where supported. A 2-month offset was included (Equation 3) to account for regimens with a 2-month intensive treatment phase followed by a continuation phase. This offset enabled regimens with a 2-month intensive phase that was identical to another regimen (e.g., HRZE/HR and HRZE) to be modeled as having the same INT value. Regimens with similar components were grouped for INT, SLP, or both parameters when necessary to maintain model stability while maximizing the ability to differentiate between regimens.

Covariates were assessed for significance on INT and SLP terms using graphical and stepwise covariate modeling during the first stage of the analysis. Stepwise covariate modeling using the likelihood ratio test was performed using respective alpha levels of 0.05 and 0.01 for forward addition and backward deletion phases. All covariates were evaluated through the inclusion of additive fixed effects on either INT or SLP. Fixed effects for continuous covariates represent the estimated effect of the covariate at the data set median value as all continuous covariates were centered for analysis. Covariate effects were reevaluated during the second stage of the analysis to confirm that the inclusion of additional data did not warrant a change in the model structure.

### Model evaluation.

Simulation-based diagnostics (i.e., visual predictive checks [VPCs]) were performed on pivotal models to evaluate model predictive performance when stratified by regimens and covariates. A total of 1000 replicates were simulated and 5th to 95th percentiles of the simulated values were overlaid with the observed values to visually assess the agreement between the model-based treatment duration-dependent relapse probabilities and the observed relapse proportions at the various treatment durations.

To obtain estimates of model precision and support comparison of the various regimens, a nonparametric bootstrap approach was employed. A total of 500 replicates of the analysis data set were generated with replacement with stratification by regimen. Model parameters were obtained through reestimation of the model on each replicated data set, with T_10_ and T_50_ estimates generated from the bootstrap model estimates at the covariate reference value to generate covariate-normalized confidence intervals. Rank orders were calculated across all regimens using the median T_10_ and T_50_ values from the bootstrap runs.

### Simulations.

Simulations were run to examine the effects of covariates on T_10_ and T_50_. Observed inoculum values in the source data set were used to generate prediction curves from the model estimates for HRZE/HR at the median baseline bacterial burden value. This was repeated for the observed baseline bacterial burden at the median inoculum value, as well as for all combinations of inoculum and baseline bacterial burden values in the data set.

### Software and hardware.

All data assembly and analysis were performed in R (33) as implemented via the RStudio environment (34). Logistic regression was done using the *glmer* function in the *lme4* package (35). Diagnostic metrics and plots were generated via the *dx* function in the *LogisticDX* (36) package and using the *ggplot2* (37) package.
