# Supplementary material for: Model-Based Meta-Analysis of Relapsing Mouse Model Studies from the Critical Path to Tuberculosis Drug Regimens Initiative Database
Source: Antimicrob Agents Chemother. 2022 Mar 15;66(3):e01793-21. doi: 10.1128/aac.01793-21 (PMC8923195; doi:10.1128/aac.01793-21)
Supplement: Supplemental file 1 — Fig. S1. Download aac.01793-21-s0001.pdf, PDF file, 1 MB [file aac.01793-21-s0001.pdf]

Supplemental Material

TABLE S-1 Study information by contributing lab

**Lab**[a]	**Study number**	**Regimens**[b] **(intensive/continuation)**	**Months treatment prior to assessment**
	1	HRZE, RMZE, HRZM	2, 3, 4
	2	HRZE/HR, HZE, HRE, HE	2, 3, 6, 9
**CSU**	3	HRZE/HR	3
	4	HRZE/HR	3
	5	HRZ/HR, RMZ/RM	3, 4, 5, 6
	6	HRZE/HR	4, 5, 6
	7	HRZE/HR	4
	8	HRZE/HR	5, 6
	9	HRZE/HR, HRZE	4
	10	HRZE	3
	11	HRZ/HR, HRZM/HRM, RZM/RM	6
**JHU**	12	HRZ/HR, RZM/RM, RZM	3, 4, 5, 6
	13	RZM/RM	3, 4
	14	HRZ/HR, RZM/RM	4, 5, 6
	15	HRZ/HR	4
	16	HRZ/HR	4, 5
	17	HRZ/HR	4, 5, 6
	18	HRZ/HR	2, 3

19	HRZ/HR	2, 3, 4
20	HRZ/HR	2, 3, 4
21	HRZE/HR, HRZE/HRZ, HRE/HR	3, 4.5, 6
22	HRZE/HR, RMZE/RM, HRZM/HRM	2, 3, 4
23	HRZ/HR	4, 6
24	HRZ/HR, RZM/RM_BIDM[c]	2.5
25	HRZ/HR, BPa, BPaL	2, 3, 4
26	HRZ/HR, BPa	2, 3
27	HRZ/HR, BPaL	2, 3
28	BPa, BPaL	2, 3

[a]CSU, Colorado State University; JHU, Johns Hopkins University.

[b]B, bedaquiline; E, ethambutol; H, isoniazid; L, linezolid; M, moxifloxacin; Pa, pretomanid; R, rifampin; Z, pyrazinamide.

[c]BIDM, moxifloxacin 100 mg/kg dosed twice daily for a total daily dose of 200 mg/kg.

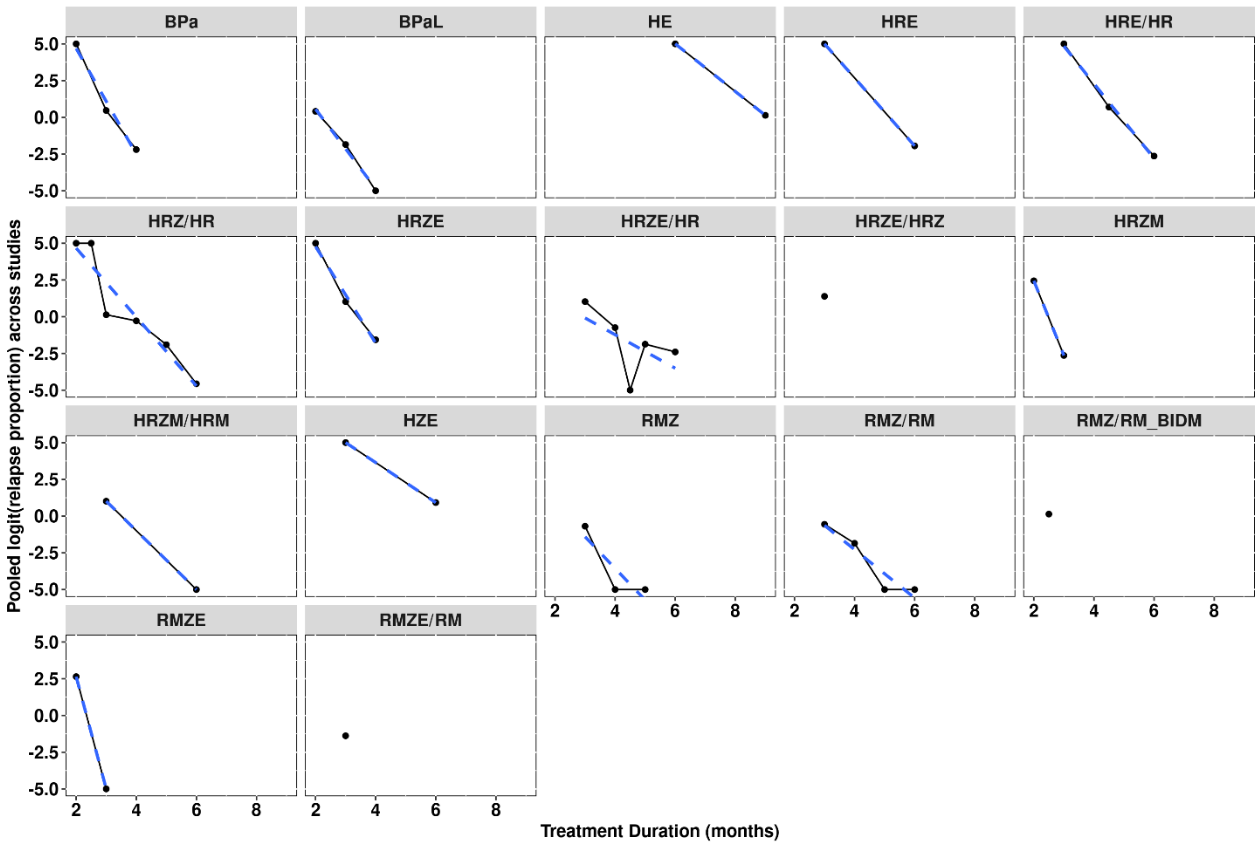

FIG S-1 Logit-transformed relapse proportion versus treatment duration by regimen. Black dots and solid lines represent the observed relapse proportion calculated across all studies. Blue dashed lines represent a linear regression fit. “RMZ/RM_BIDM” denotes a version of the RMZ/RM regimen where moxifloxacin was administered at 100 mg/kg twice daily for a total daily dose of 200 mg/kg. M, moxifloxacin; R, rifampin; Z, pyrazinamide.

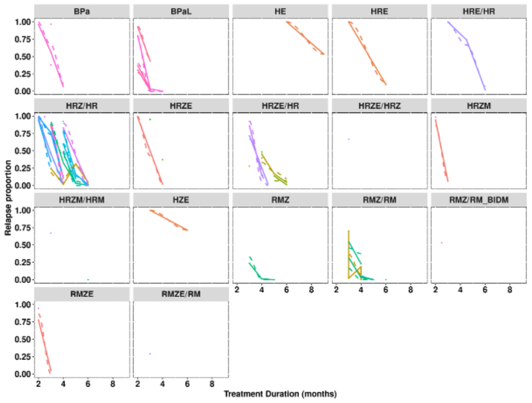

(a)

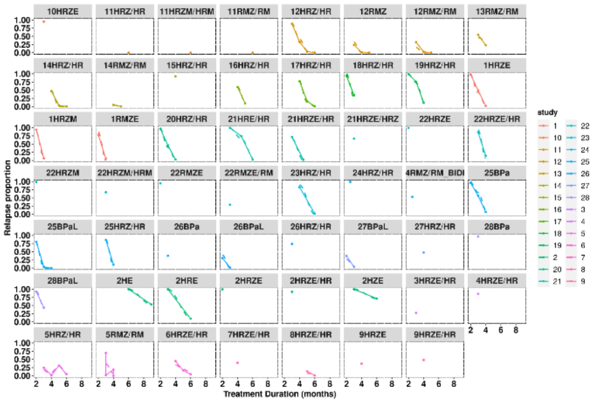

(b)

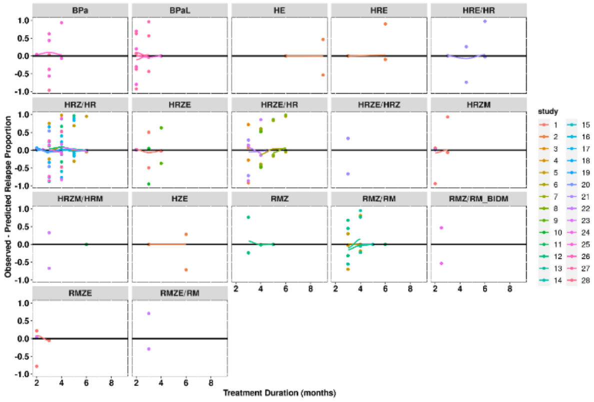

(c)

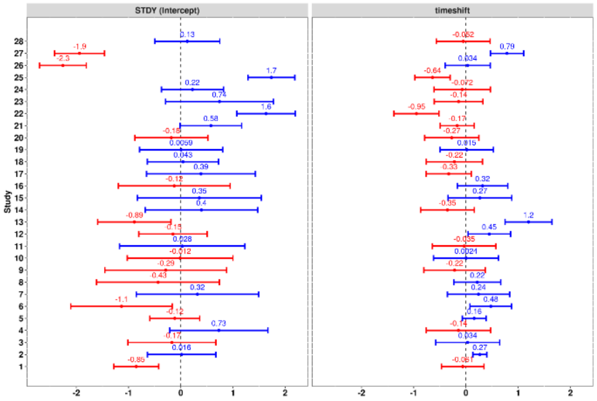

(d)

FIG S-2 Goodness-of-fit plots for the final model. Goodness-of-fit plots for the final model: a) relapse proportion (observed [points] with overlaid predictions [solid lines]) versus time stratified by regimen, b) relapse proportion (observed [points] with overlaid predictions [solid lines]) versus time stratified by study and regimen, c) observed minus predicted relapse proportion versus time stratified by regimen and study, and d) forest plot of random effects on intercept (INT) and slope (SLP). Overall, the model is able to describe the data well. “RMZ/RM_BIDM” denotes a version of the RMZ/RM regimen where moxifloxacin was administered at 100 mg/kg twice daily for a total daily dose of 200 mg/kg. M, moxifloxacin; R, rifampin; Z, pyrazinamide.

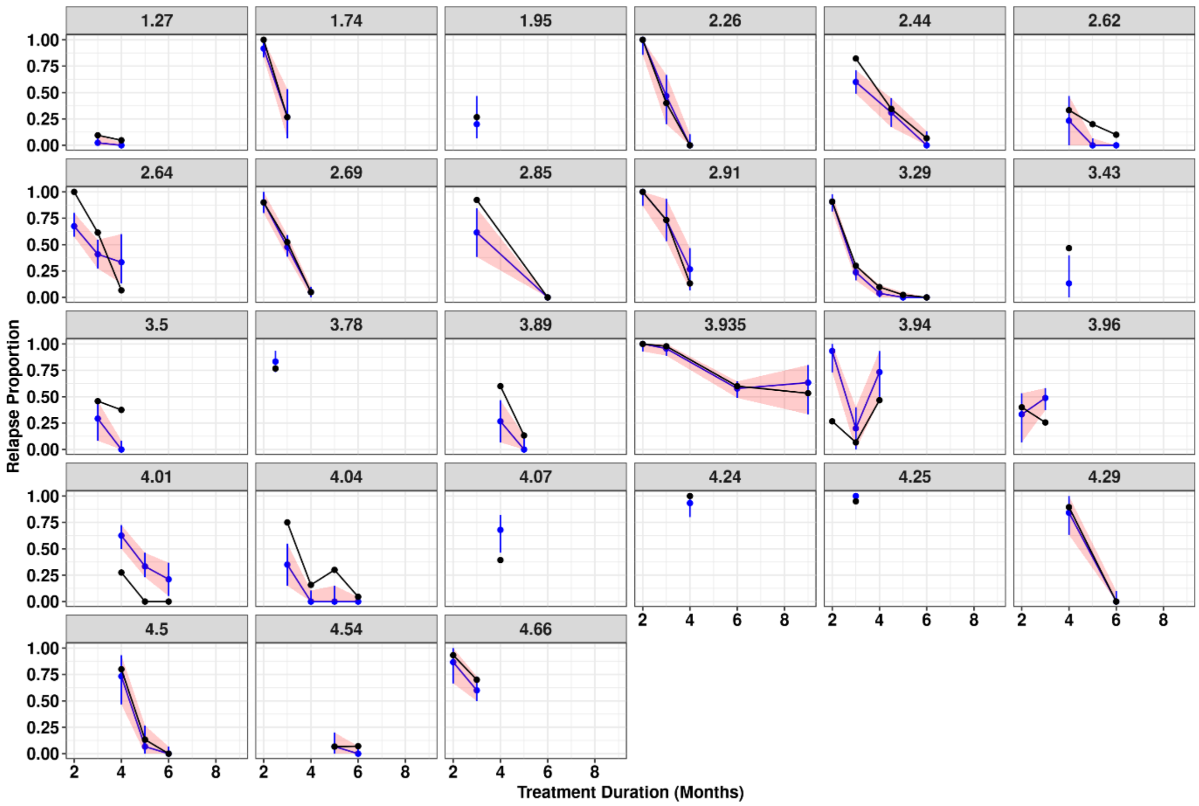

FIG S-3 Visual predictive check for the final model – stratified by inoculum (Log_{10} CFU). Black dots and solid lines represent the observed relapse proportion. Blue dots and solid lines represent the median prediction from the final model. The red shaded area represents the 90% prediction interval.

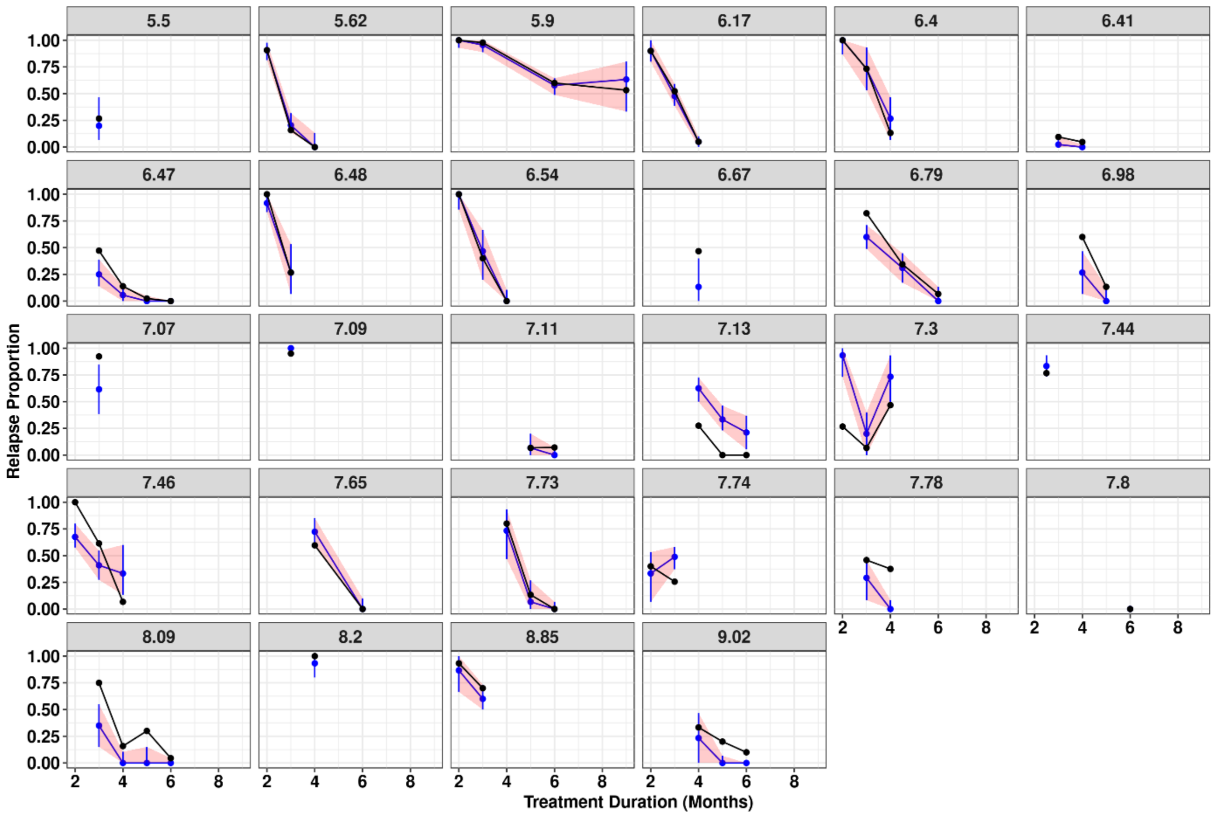

FIG S-4 Visual predictive check for final model – stratified by baseline bacterial burden (Log_{10} CFU). Black dots and solid lines represent the observed relapse proportion. Blue dots and solid lines represent the median prediction from the final model. The red shaded area represents the 90% prediction interval.

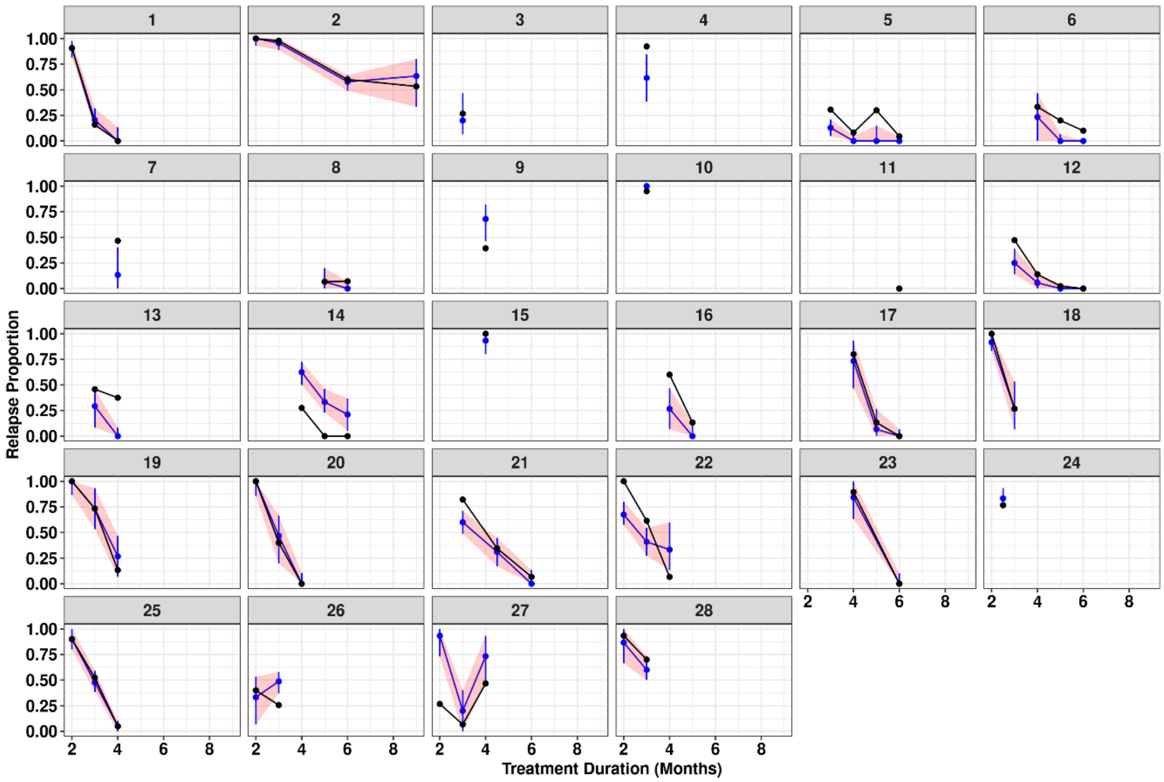

FIG S-5 Visual predictive check for final model – stratified by study. Black dots and solid lines represent the observed relapse proportion. Blue dots and solid lines represent the median prediction from the final model. The red shaded area represents the 90% prediction interval.
